# Dataset on biodiversity conservation among Swiss farmers: A focus on landscape features

**DOI:** 10.1016/j.dib.2026.113344

**Published:** 2026-10-12

**Authors:** Christian Ritzel, Antonia Kaiser, Yanbing Wang, Gabriele Mack

**Affiliations:** a Economic Modelling and Policy Analysis, Agroscope, Tänikon 1, 8356, Ettenhasuen, Switzerland; b University of Basel, Social Transitions Research Group, 4051 Basel, Switzerland

**Keywords:** Landscape elements, Self-efficacy, Personal norms, Social norms, Policy priority, Farmer self-identity

## Abstract

From June to August 2023, we conducted a combined paper-and-pencil and online survey. We sent a questionnaire by post to 2000 farmers in the German- and French-speaking language regions of Switzerland. We also sent them a link to an online version of the survey programmed in the survey tool Tivian. The response rate was 44%, resulting in 882 completed surveys. We used stratified sampling by agricultural zone and farm type. The farmers’ contact details were provided by the Swiss Federal Office for Agriculture, which maintains a database of approximately 48,000 farms receiving direct payments, accounting for over 99% of all Swiss farms. The survey contained questions on the implementation status and subjective importance of landscape features (e.g., field margins on arable land, hedges, single trees), sociodemographic characteristics, information and extension sources, self-efficacy in biodiversity conservation, personal and social norms in biodiversity conservation, farmer roles, policy priorities, and the importance of income sources.

Specifications Table

**Table:** 

Subject	Social Sciences
Specific subject area	Agricultural Economics and Agricultural Sociology
Type of data	CSV file, DTA file (STATA) and codebook (PDF)
Data collection	In 2023, we conducted a survey among 2000 Swiss farmers. We used stratified sampling by agricultural zone and farm type. The farmers were sent a paper-and-pencil survey by postal mail as well as a link to an online version of the survey programmed in the survey tool Tivian. The survey contained questions on the implementation status of landscape features, the importance of implementation aspects, sociodemographic and farm characteristics, information and extension sources used, self-efficacy in biodiversity conservation, personal and social norms in biodiversity conservation, self-identity, policy priorities, and the importance of income sources.
Data source location	Institution: Agroscope City/Town/Region: Ettenhausen, Tänikon Country: Switzerland
Data accessibility	Repository name: Zenodo Data identification number: 10.5281/zenodo.20628284 Direct URL to data: https://zenodo.org/records/20628284 Instructions for accessing these data: The data are freely accessible via the Zenodo repository.
Related research article	Parts of the data were used in the following article: C. Ritzel, A. Kaiser, Y. Wang, G. Mack, The role of social and personal norms in biodiversity conservation: A segmentation of Swiss farmers. J. Environ. Manag., 377 (2025) 124,605. https://doi.org/10.1016/j.jenvman.2025.124605.

## 1 Value of the Data

• Overall, the survey data from 882 Swiss farms closely represents the population of Swiss farms in terms of farm type, agricultural zone, and utilized agricultural area.
• The data collected across the German-speaking and French-speaking language regions enable an understanding of the cultural differences within Switzerland. Multi-language surveys can also be used in other countries.
• The data contain items on farmers’ social and personal norms, identities, and values regarding biodiversity conservation and the observed behavior in terms of the implementation of landscape features. This allows researchers to link farmers’ behavioral characteristics to their actual pro-environmental behavior.
• The data can be reused for new research questions, replication analyses, cross-country comparisons, and meta-analyses. For example, researchers could use data on farmers’ socio-economic characteristics to predict their policy priorities. Furthermore, the data can be used to address new research questions, such as whether different farmer types have different policy priorities by linking the farmer identity construct with farmers’ policy priorities.
• The dataset’s focus on landscape features, combined with rich information on farm and farmer characteristics, enables policy-relevant analyses of both the adoption of specific biodiversity measures and the underlying factors that drive or hinder their implementation.

## 2 Background

The agricultural sector plays a major role in global anthropogenic biodiversity loss through land-use change, overexploitation of natural resources such as groundwater aquifers, greenhouse gas emissions from livestock production, and pesticide pollution [1]. Agricultural subsidy systems can create misaligned incentives that unintentionally encourage environmentally harmful practices [2]. In the case of Switzerland, Gubler et al. [3] hypothesized that the current direct payment system—particularly food security payments—is negatively associated with the implementation of landscape features such as hedges, single trees, and field margins on arable land. These landscape features serve as important biodiversity hotspots within and between crops in intensively managed agricultural landscapes [4]. Therefore, assessing the determinants of the on-farm implementation of landscape features is crucial.

The purpose of this article is to provide an overview of a survey designed to capture biodiversity conservation among Swiss farmers with a focus on landscape features, as well as the variables and their summary statistics. It further describes the data collection process and outlines the dataset’s limitations. The data article includes the full dataset, the survey, and the codebook. This dataset can be reused to answer new research questions, replicate analyses, conduct cross-country comparisons, and perform meta-analyses.

## 3 Data Description

This article includes links to the codebook, which describes all variables, and to the data files in Stata (.dta), Excel (.xlsx) and CSV (.csv) formats. The purpose of the survey was to provide a comprehensive overview of landscape features implemented on Swiss farms, as well as farmers’ norms and attitudes toward biodiversity conservation and Swiss agricultural policy. The survey data are organized into four sections. The first part records the farmers’ sociodemographic and farm characteristics (Section 3.1). The second part refers to the status of landscape features on the farms, as well as the importance of aspects related to the implementation, non-implementation, and removal of landscape features (Section 3.2). The third part includes data on the farmers’ personal and social norms, skills, and roles (Section 3.3). The fourth part comprises the farmers’ assessment of Swiss agricultural policy (Section 3.4). The variables are coded with a running number (i.e., v1, v2, etc.).

### 3.1 Sociodemographic and farm characteristics

Table 1 shows the summary statistics of the variables related to farmers’ sociodemographic characteristics and farm characteristics.

**Table 1:** Summary statistics of the variables related to farmers’ sociodemographic characteristics and farm characteristics.

Variable	Scale	Freq. (%)	Mean	SD	Min.	Max.
Language region	Binary					
	1 = German-speaking	83.7				
	2 = French-speaking	16.3				
Q1 Participation in quality assurance and certification programs	Binary					
None	Yes = 1	14.6				
	No = 2	84.7				
IP SUISSE	Yes = 1	55.1				
	No = 2	44.2				
Organic	Yes = 1	21.1				
	No = 2	78.2				
Demeter	Yes = 1	1.1				
	No = 2	98.2				
Other	Yes = 1	12.9				
	No = 2	86.4				
Q2 Number of years a farmer had been running the farm	Continuous (years)		18.9	11.4	0.0	51.0
Q3 Farmers operating their farms full-time or part-time	Binary					
	1 = Full-time	75.1				
	2 = Part-time	24.1				
Q4 Educational level	Nominal					
	1 = Practical experience	5.6				
	2 = In training / apprenticeship	0.2				
	3 = Federal vocational training (EBA)	4.4				
	4 = Federal certificate of competence (EFZ)	44.0				
	5 = Professional examination	11.8				
	6 = Master’s examination	20.7				
	7 = Higher college	4.4				
	8 = Bachelor’s degree / master’s degree or higher	4.5				
	9 = other education	3.5				
Farmers’ age	Continuous (years)		48.7	10.5	21.0	79.0

The percentage of farms located in the German-speaking (83.7%) and French-speaking (16.3%) regions of Switzerland (v1). Information on the language region was not directly collected in the survey but was based on the farm’s postal address. The percentage of farmers who participated in quality assurance and certification programs as a binary choice (yes or no), including missing values (NA). The farmers could indicate whether they participated in none (v2), IP SUISSE^1^ (v3), organic (v4), Demeter (v5), or other (v6) programs. If the farmers participated in other quality assurance and certification programs, they could indicate the programs using a text box (v7). For all variables, we observed 0.7% missing values. In total, 14.6% of the farmers did not participate in any quality assurance or certification programs, whereas 84.7% participated in at least one. Participation in IP Suisse was reported by 55.1% of the farmers. Organic certification was held by 22.1% of the farmers. Only 1.1% of the farmers were certified under the Demeter standard. Additionally, 12.9% participated in other quality assurance or certification programs. Variable v8 refers to the number of years a farmer had been running the farm. Its mean value was 18.9 years, its standard deviation (SD) was 11.4 years, its minimum value was 0.0, and its maximum value was 51.0 years. There were 11 missing values in the data, resulting in 872 observations. The binary variable v9 indicates whether a farmer operated the farm full-time (75.1%) or part-time (24.1%). A full-time operation is defined as deriving >50% of a farmer’s income from farming activities, while a part-time operation derives as 50% or less of a farmer’s income from farming activities. Missing values are indicated by NA (0.8%). The nominal scaled variable v10 reflects a farmer’s educational level. Values 1 to 8 represent pre-defined educational levels. Value 9 represents other education, and NA represents missing values. Other education levels could be indicated by farmers in a text box (v11). Overall, 5.6% of the farmers indicated that they had practical experience, 0.2% reported being in training or apprenticeship, and 4.4% had obtained a federal vocational certificate (EBA). A total of 44.0% held a federal certificate of competence (EFZ). Among the farmers, 11.8% had completed a professional examination, 20.7% had passed a master’s examination, 4.4% had obtained a higher college degree, and 4.5% held a bachelor’s or master’s degree. Additionally, 3.5% reported having other types of education. Overall, 0.8% of the observations were missing. The mean of the continuous variable farmer’s age (v12) was 48.7 years, with a standard deviation (SD) of 10.5 years, a minimum value of 21.0 years, and a maximum value of 79.0 years. There were 12 missing values in the data, resulting in 871 observations.

### 3.2 Landscape features on the farms

Fig. 1 shows the percentage distribution of the farmers’ responses regarding the following landscape features: (a) field margins on arable land, (b) litter meadows, (c) hedges, (d) ditches, (e) unpaved roads, (f) ruderal areas, (g) dry-stack walls, (h) high-trunked fruit trees, (i) nut trees, (j) sweet chestnut trees, (k) site-specific single trees and avenues, (l) distinctive individual trees, and (m) other trees. The possible values for the nominal scaled variable “implementation status” are “currently implemented,” “never implemented,” and “removed.” Missing values are indicated by NA. The farmers rated the “aspects of importance” with regard to “currently implemented,” “never implemented,” or “removed” for the following eight ordinal scaled variables: “aesthetic,” “expected impact on biodiversity,” “feasibility,” “financial,” “societal demands,” “own knowledge,” “external advice,” and “other.” These ordinal scaled variables are rated on a scale of 1 to 3, where 1 is “very important,” 2 is “important,” and 3 is “not important.” The farmers further indicated any unmentioned aspects using a text box. Missing values are indicated by NA.

**Fig. 1:**
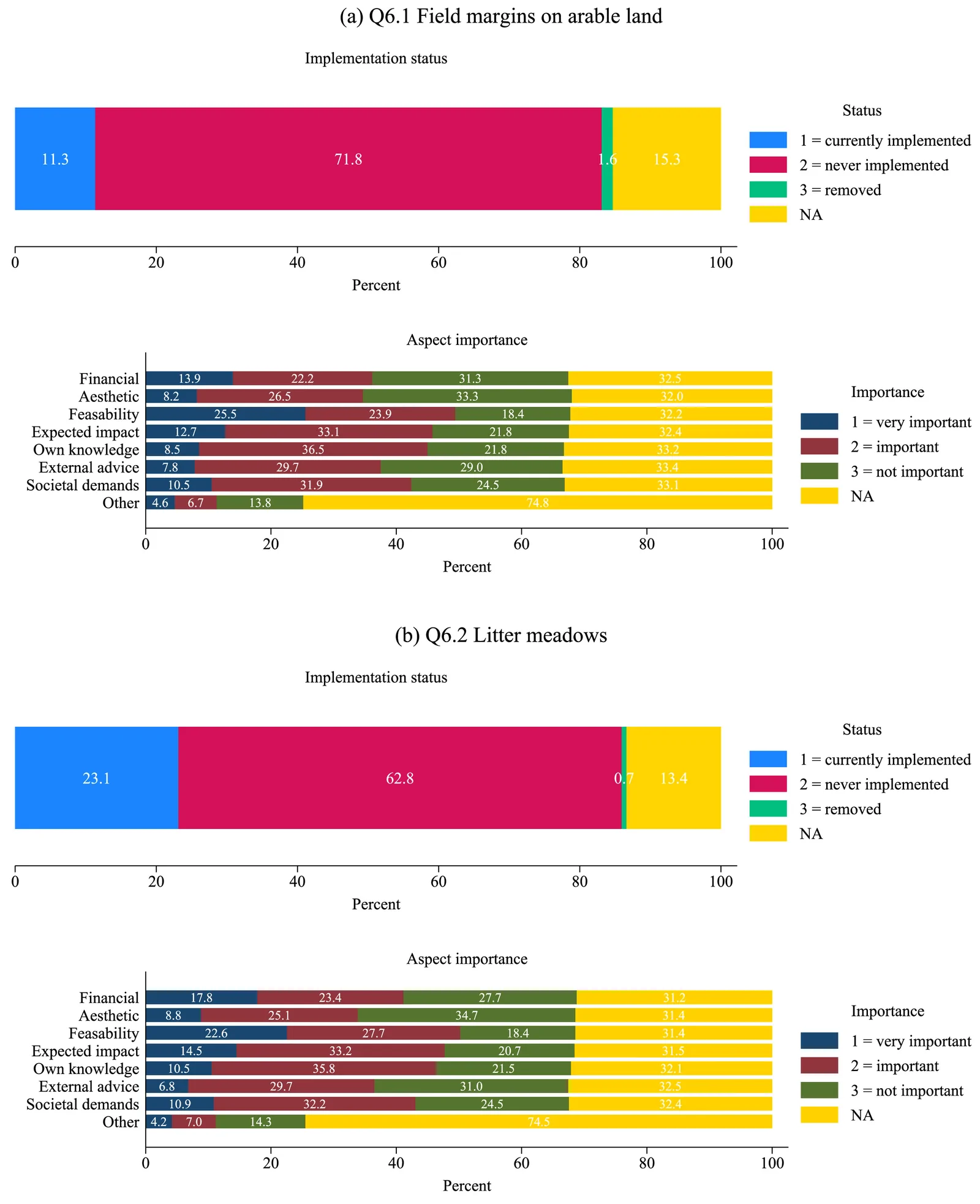

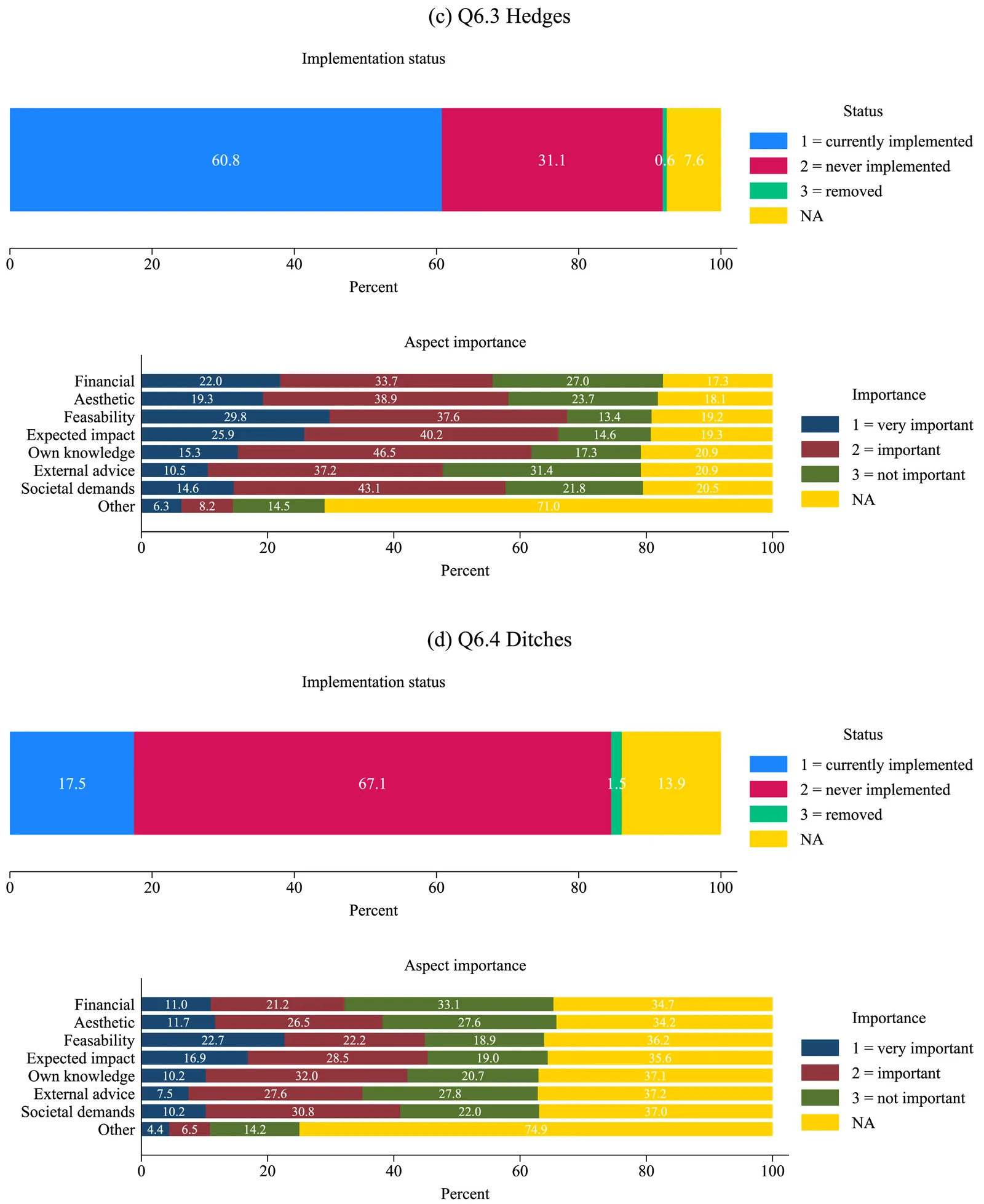

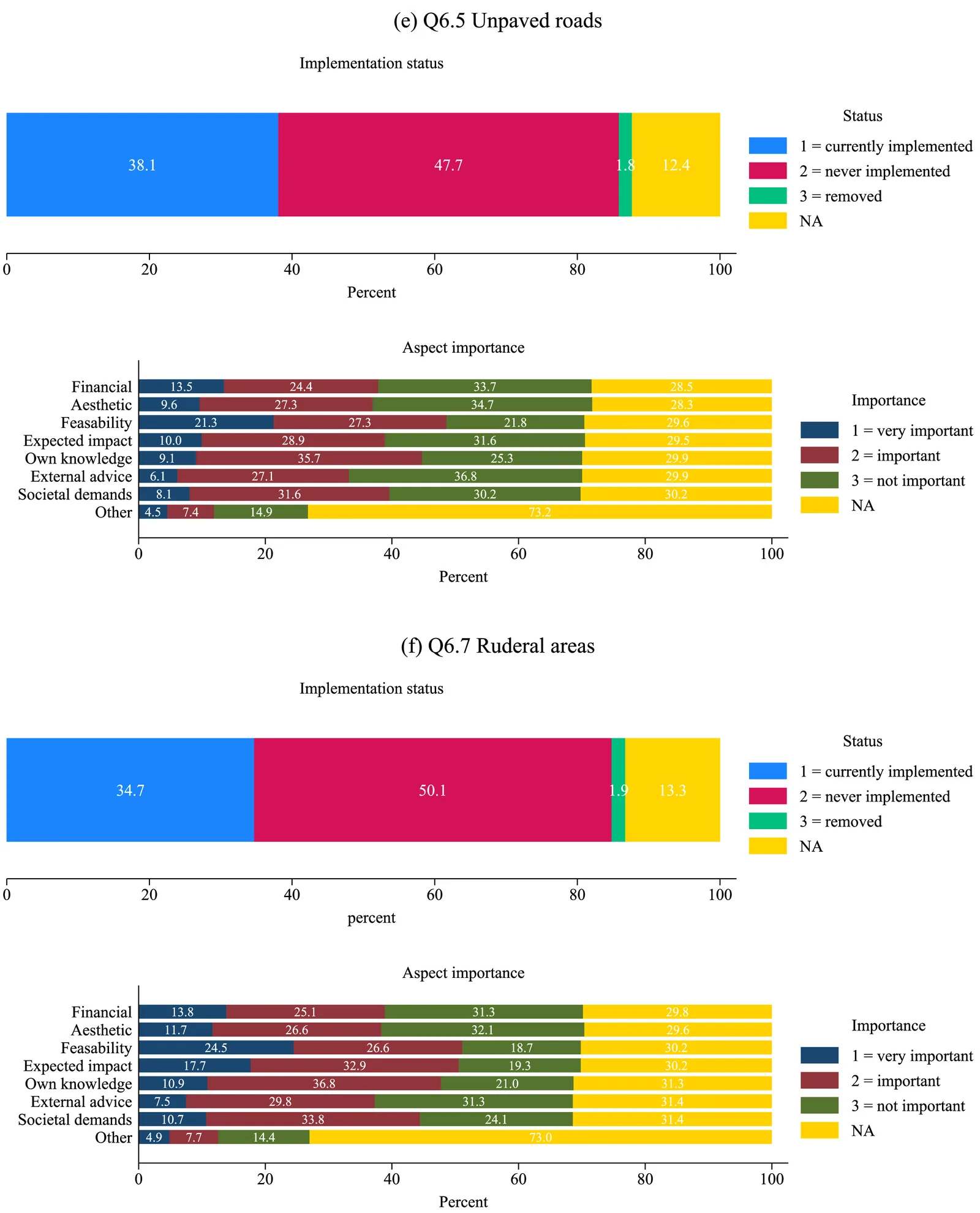

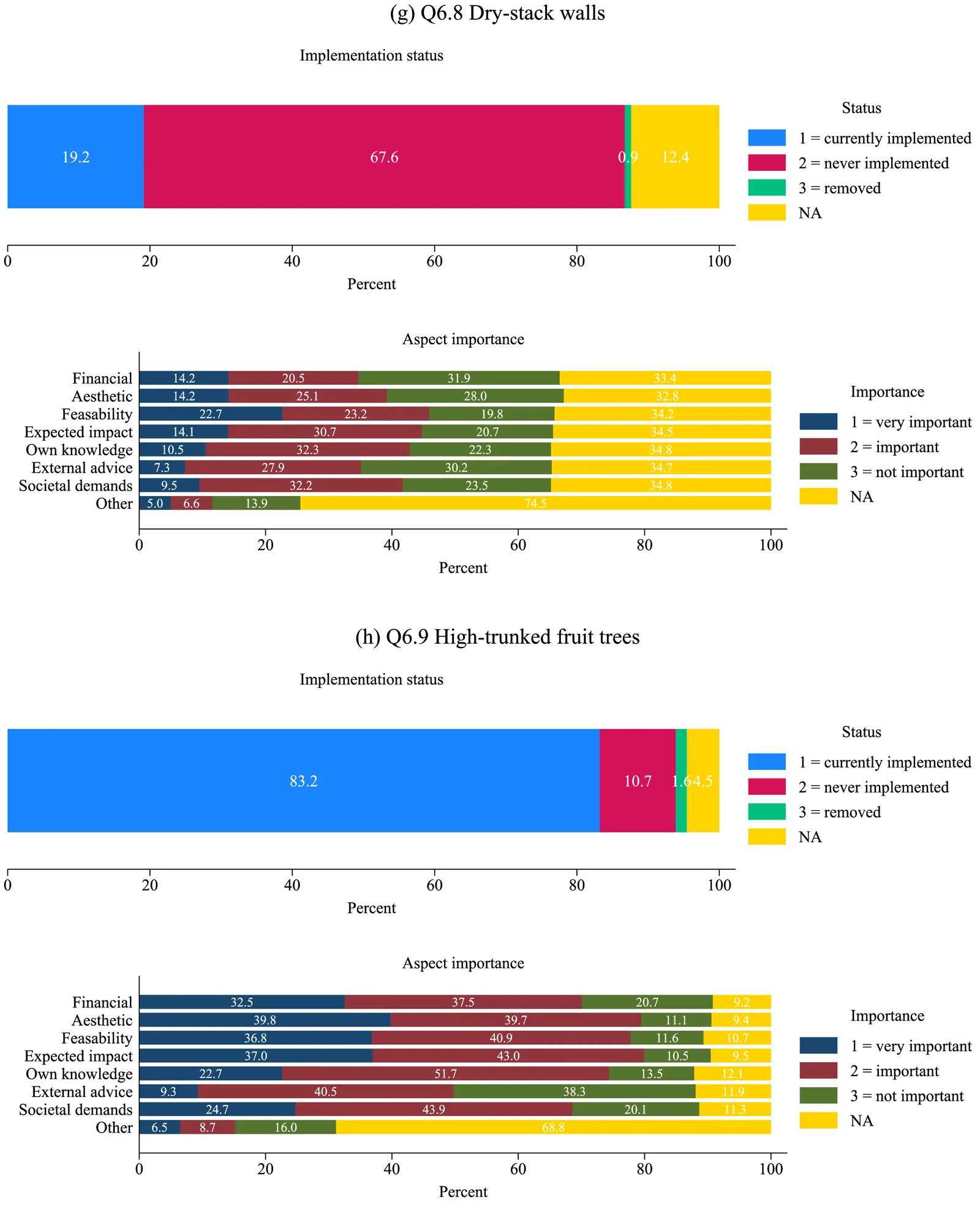

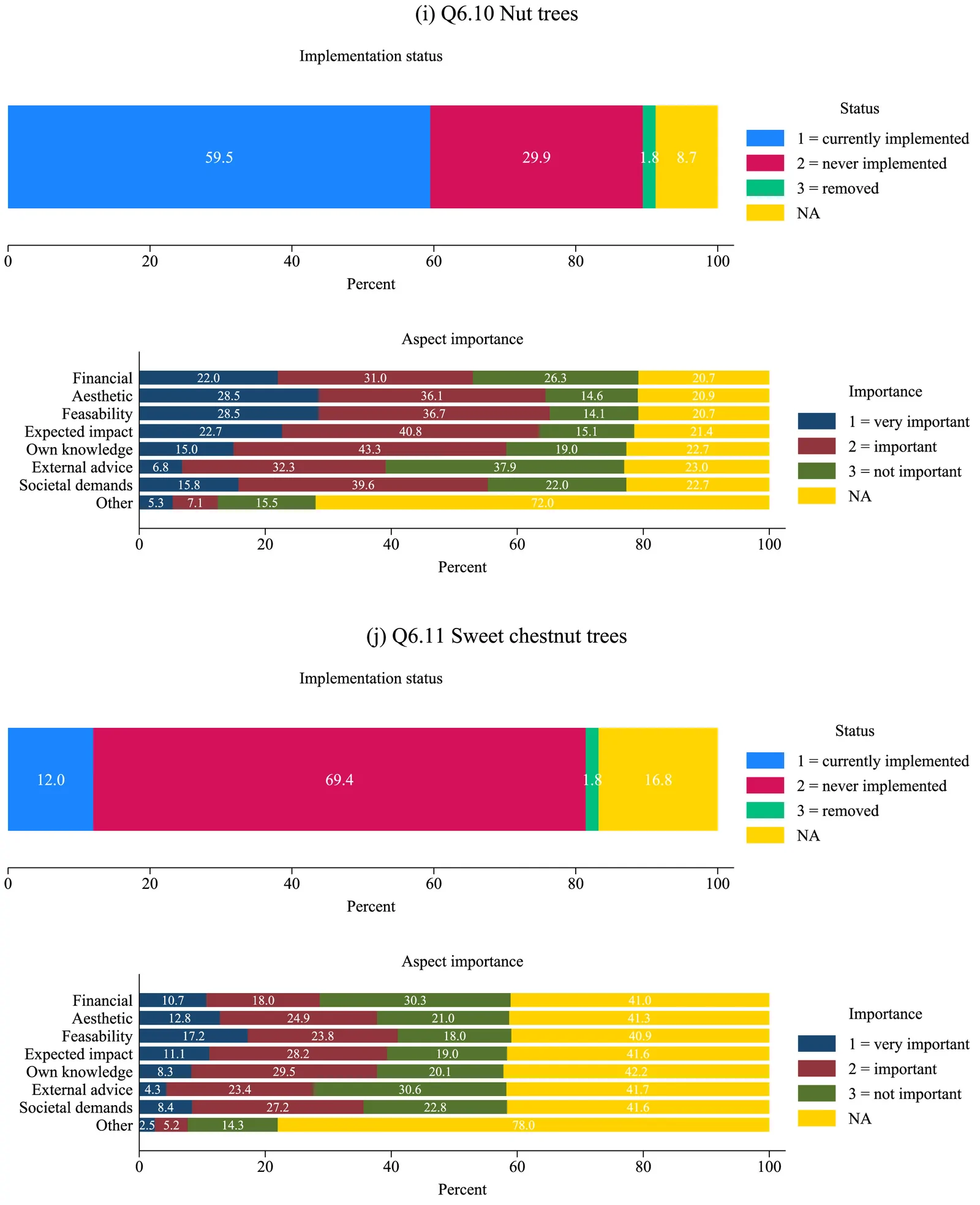

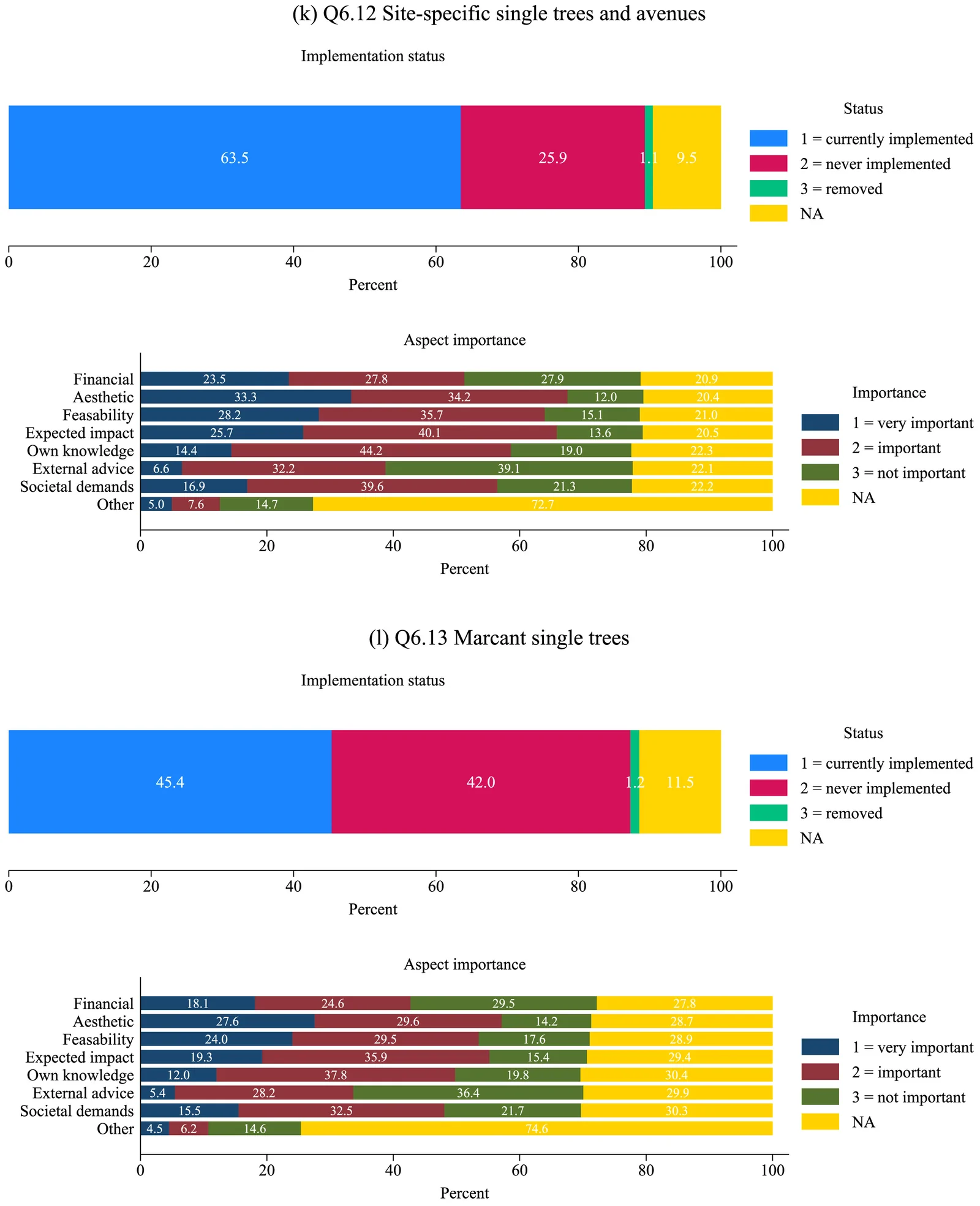

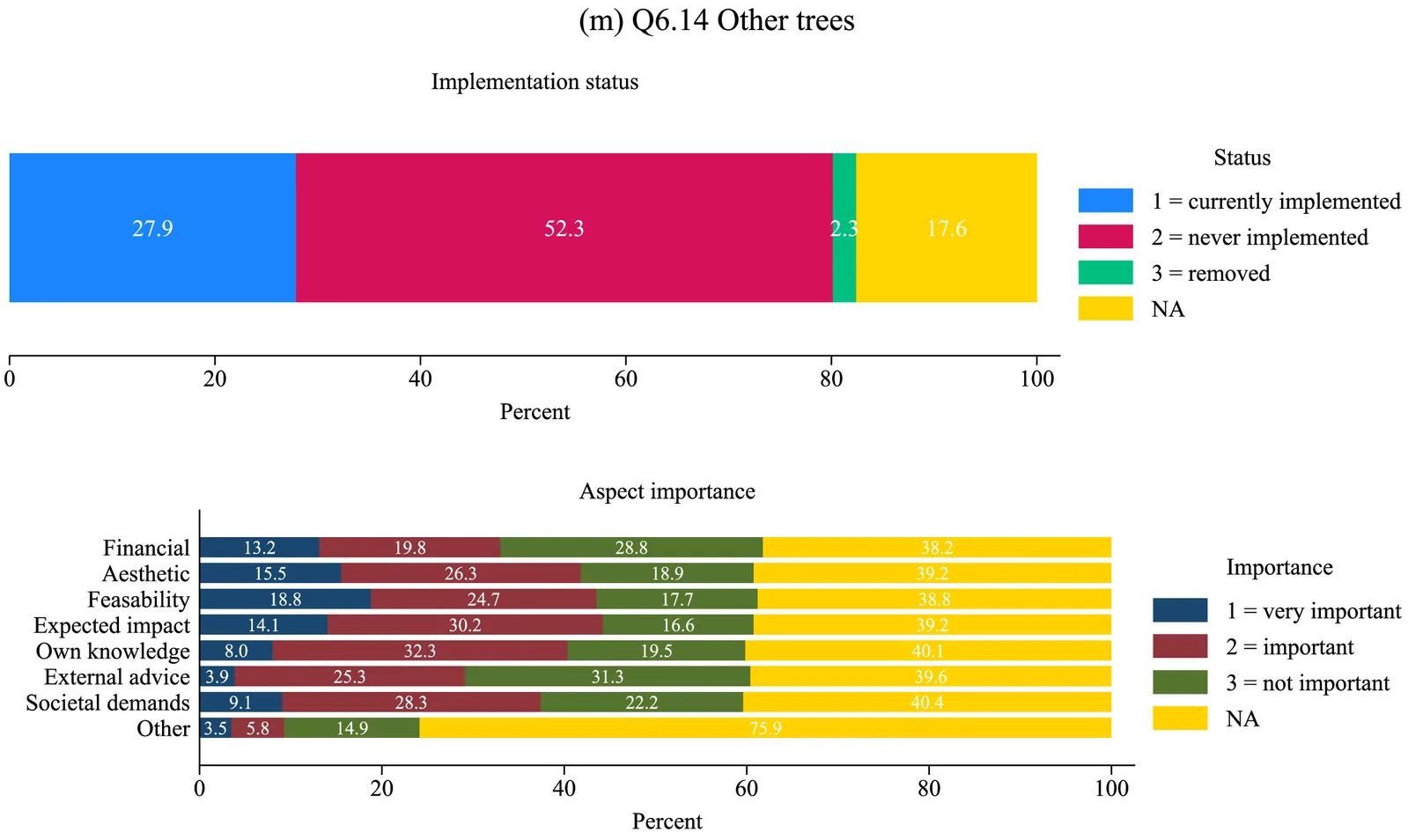
Percentage distribution of the farmers’ responses regarding implementation status and aspect importance for the landscape features: (a) field margins on arable land, (b) litter meadows, (c) hedges, (d) ditches, (e) unpaved roads, (f) ruderal areas, (g) dry-stack walls, (h) high-trunked fruit trees, (i) nut trees, (j) sweet chestnut trees, (k) site-specific single trees and avenues, (l) distinctive individual trees, and (m) other trees.

Overall, each landscape feature spanned 10 variables, with the first being the implementation of the landscape feature, followed by 8 pre-defined aspects regarding the landscape feature, as well as one variable for other aspects defined by the farmer. For example, the first landscape feature, “(a) field margins on arable land,” covers the following variables: v13 “implementation status of field margins,” v14 “financial,” v15, “aesthetic,” v16 “feasibility,” v17 “expected impact,” v18 “own knowledge,” v19 “external advice,” v20 “societal demands,” and v21 “other.” Other aspects could be indicated by farmers in a text box (v22). The last landscape feature “(m) other trees” covers the following variables: v133 “implementation status of other trees,” v134 “financial,” v135 “aesthetic,” v136 “feasibility,” v137 “expected impact,” v138 “own knowledge,” v139 “external advice,” v140 “societal demands,” and v141 “other.” The other aspects indicated by the farmers in the text box are covered by variable v142.

Across all landscape features, between 11.3% (field margins on arable land) and 83.2% (high‑stem fruit trees) of the farmers indicated that a landscape feature was currently implemented, with the remaining responses mainly indicating non‑implementation (i.e., “never implemented”). The share of responses in the category “removed” ranged from 0.6% (hedges) to 2.3% (other trees). Missing values for implementation status accounted for 4.5% (high‑trunked fruit trees) to 17.6% (other trees) of the responses.

Regarding the “aspect importance” of the landscape features, the farmers’ responses were predominantly distributed between the categories “very important” and “important.” Nevertheless, the landscape features indicated by the farmers in the aspect importance categories varied: 2.5% (other for sweet chestnut trees, panel j) to 39.8% (high-trunked fruit trees, panel h) of the farmers classified an aspect as “very important,” while 5.2% (other for sweet chestnut trees, panel j) to 51.7% (own knowledge for high‑trunked fruit trees, panel h) rated it as “important.” The share of responses indicating “not important” ranged from 13.8% (other for field margins on arable land, panel a) to 39.1% (external advice for site-specific single trees and avenues, panel k). Missing values accounted for 9.4% (aesthetic for high‑trunked fruit trees, panel h) to 78.0% (category “other” for sweet chestnut trees, panel j) of the responses.

### 3.3 Personal and social norms, skills, and farmers’ roles

The farmers’ personal and social norms in biodiversity conservation were measured using items developed by Cialdini et al. [5] on a 7-point Likert scale. Fig. 2 visualizes the percentage distribution of the farmers’ responses, ranging from “1 = strongly disagree” to “7 = strongly agree,” to the four items that capture their personal and social norms regarding biodiversity conservation. One item referred to the personal norm (v143), two items covered injunctive norms (v144 “family,” and v145 “acquaintances”), and one item depicted the descriptive norm (v146). Missing values are indicated by NA.

**Fig. 2:**
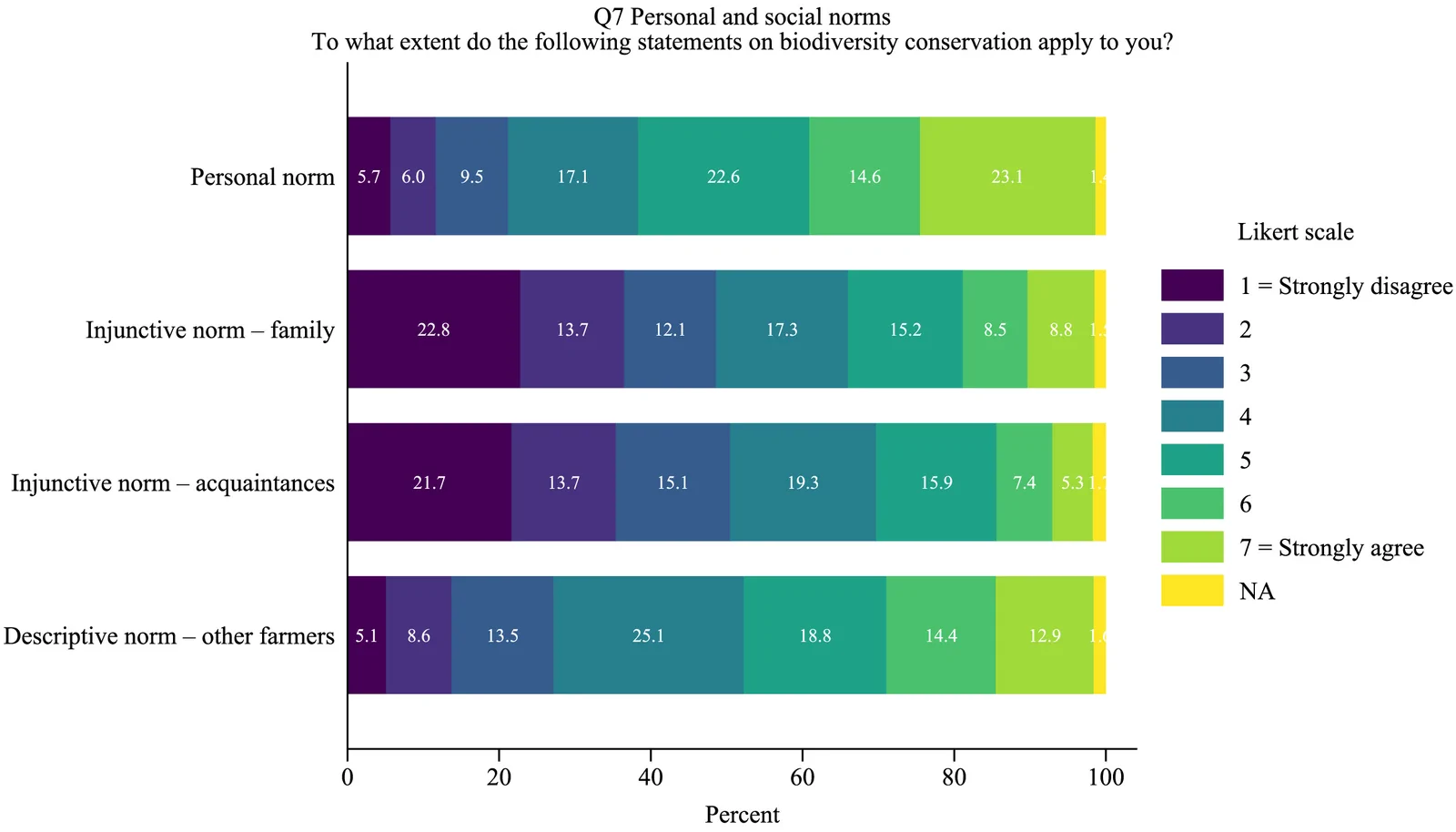
Percentage distribution of the farmers’ responses to the four items that captured their personal and social norms regarding biodiversity conservation.

For the item personal norms, 5.7% of the farmers selected “1 = strongly disagree,” whereas 23.1% indicated ”7 = strongly agree.” For the items injunctive norms, the share of responses for “1 = strongly disagree” and “7 = strongly agree” was higher for family (1 = 22.8%; 7 = 8.8%) than for acquaintances (1 = 21.7%; 7 = 5.3%). With regard to the item descriptive norms, 5.1% of the farmers indicated “1 = strongly disagree” and 12.9% indicated “7 = strongly agree.” Missing values ranged between 1.4% (item personal norms) and 1.7% (item social norm – acquaintances).

Items related to farmers’ self-efficacy were adopted from Bandura [6]. These three items were measured using a 7-point Likert scale and refer to “personal skills in biodiversity conservation” (v147), “prevention of damage to biodiversity caused by production” (v148), and “overcoming difficulties in biodiversity conservation” (v149). Missing values are indicated by NA. Fig. 3 shows the percentage distribution of the farmers’ responses to these three items, ranging from “1 = strongly disagree” to “7 = strongly agree.”

**Fig. 3:**
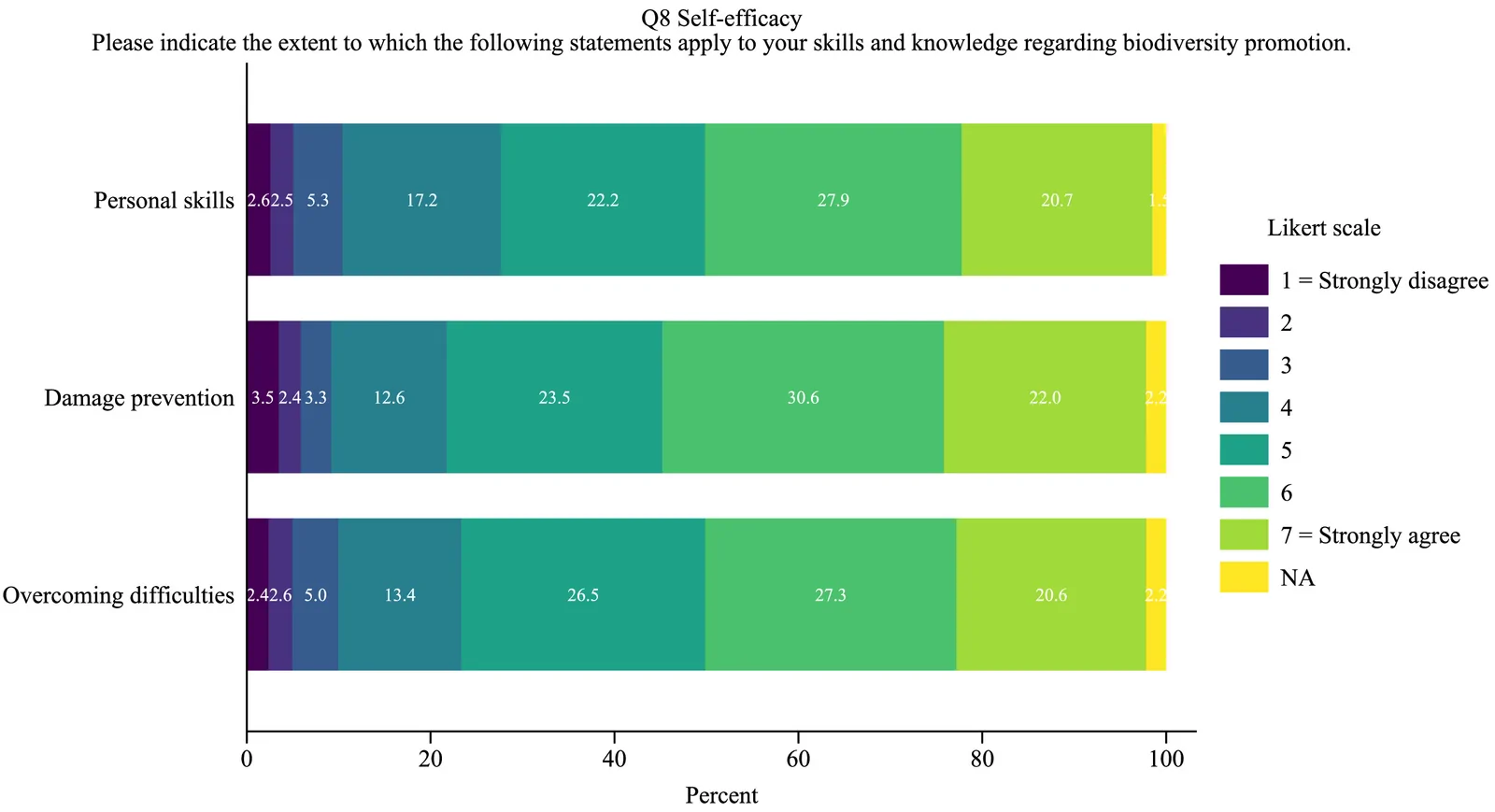
Percentage distribution of the farmers’ responses to the three self-efficacy items.

For the item personal skills, 2.6% of the farmers selected “1 = strongly disagree,” while 20.8% selected “7 = strongly agree.” Similarly, for the items damage prevention and overcoming difficulties, only 3.5% and 2.4%, respectively, selected “1 = strongly disagree,” whereas 22.0% and 20.6%, respectively, selected “7 = strongly agree.” Missing values accounted for 1.5% for the item personal skills and 2.2% for the items damage prevention and overcoming difficulties.

For optimist items 1, 2, and 3, the share of farmers who selected “1 = strongly disagree” was 2.9%, 22.8%, and 0.9%, respectively, whereas “7 = strongly agree” was selected by 20.5%, 17.5%, and 34.5%, respectively. For productivist items 1 and 2, 25.1% and 19.5% of the farmers selected “1 = strongly disagree,” while 12.1% and 8.3% selected “7 = strongly agree,” respectively. For the caretaker items 1, 2, and 3, the percentage of farmers indicating “1 = strongly disagree” was 2.0%, 0.1%, and 2.3%, respectively, while “7 = strongly agree” was chosen by 38.5%, 76.3%, and 28.5%, respectively. For conservative items 1 and 2, 3.1% and 7.9% of the farmers selected “1 = strongly disagree,” whereas 9.5% and 7.5% selected “7 = strongly agree,” respectively. For the item reflecting farmer as caretaker and productivist, 3.2% indicated “1 = strongly disagree” and 22.0% selected “7 = strongly agree.” Missing values ranged between 1.3% and 2.3%.

Fig. 4 depicts the percentage of information and extension services used by farmers, presented as a binary choice (yes or no), including missing values (NA). The sources were colleagues (v150), cantonal extension services (v151), extension projects (v152), research institutions (v153), nature conservation organizations (v154), agricultural journals (v155), own research (v156), none (v157), and other sources (v158). Other information and extension sources could be indicated by farmers in a text box (v159).

**Fig. 4:**
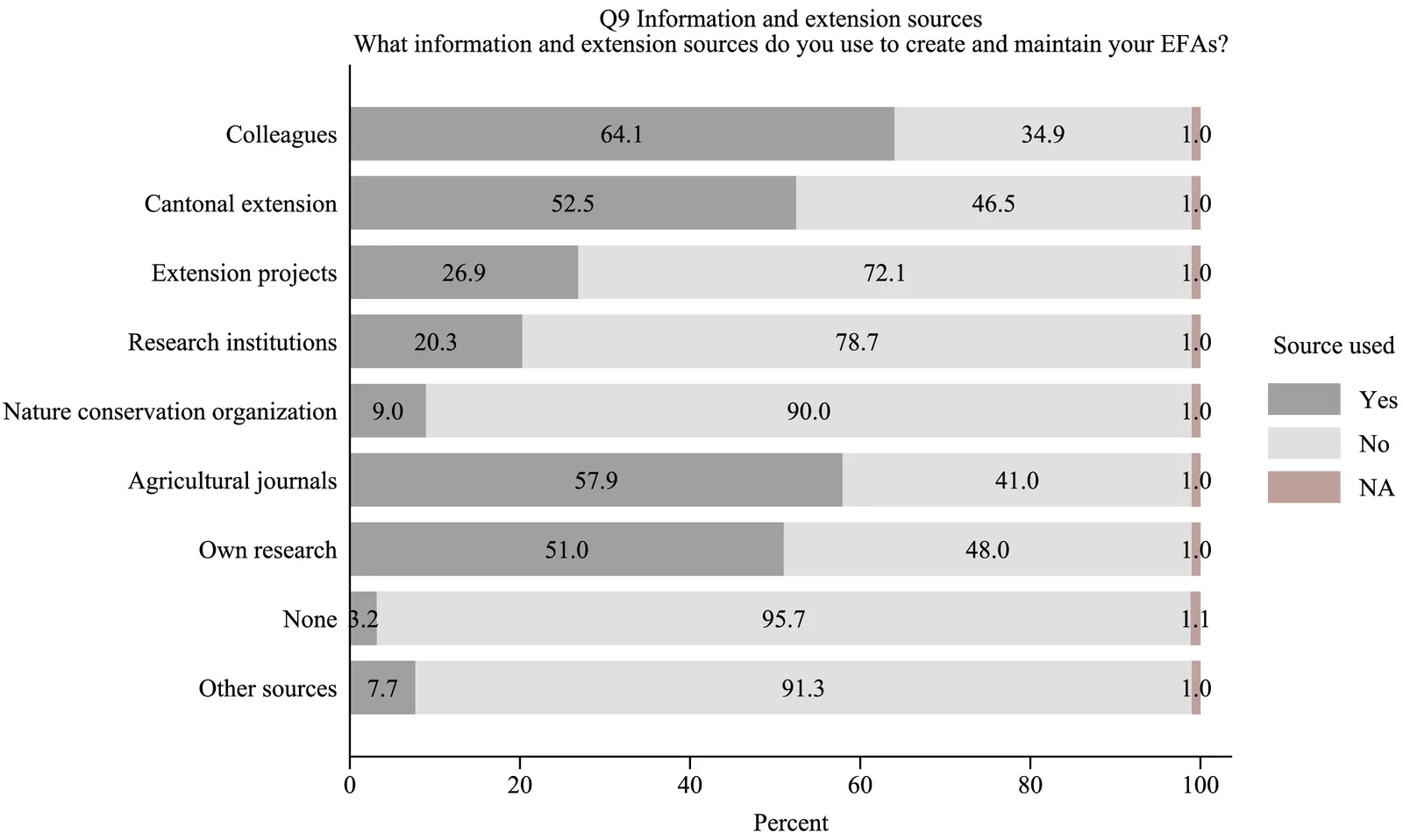
Percentage of information and extension services used or not used by the farmers.

Of the farmers, 64.1% indicated that their colleagues were their information and extension sources. Cantonal extension and extension projects were selected by 52.5% and 26.9% of the farmers, respectively, and research institutions were selected by 20.3%. Only 9.0% of the farmers used nature conservation organizations as an information and extension source. Higher shares were observed for agricultural journals (57.9%) and own research (51.0%). Only 3.2% used no information or extension sources, and other sources were only used by 7.7% of the farmers. Missing values ranged between 1.0% and 1.1%.

Fig. 5 presents the percentage distribution of the farmers’ responses to 11 items measuring their farmer self-identity on a 7-point Likert scale ranging from “1 = strongly disagree” to “7 = strongly agree.” This set of items was adapted from Cullen et al. [7]. Three items refer to an optimistic farmer (v160, v161, v162), two to a productivist farmer (v163, v164), three to a caretaker farmer (v165, v166, v167), two to a conservative farmer (v168, v169), and one to a farmer with both a caretaker and a productivist self-identity (v170). Missing values are indicated by NA.

**Fig. 5:**
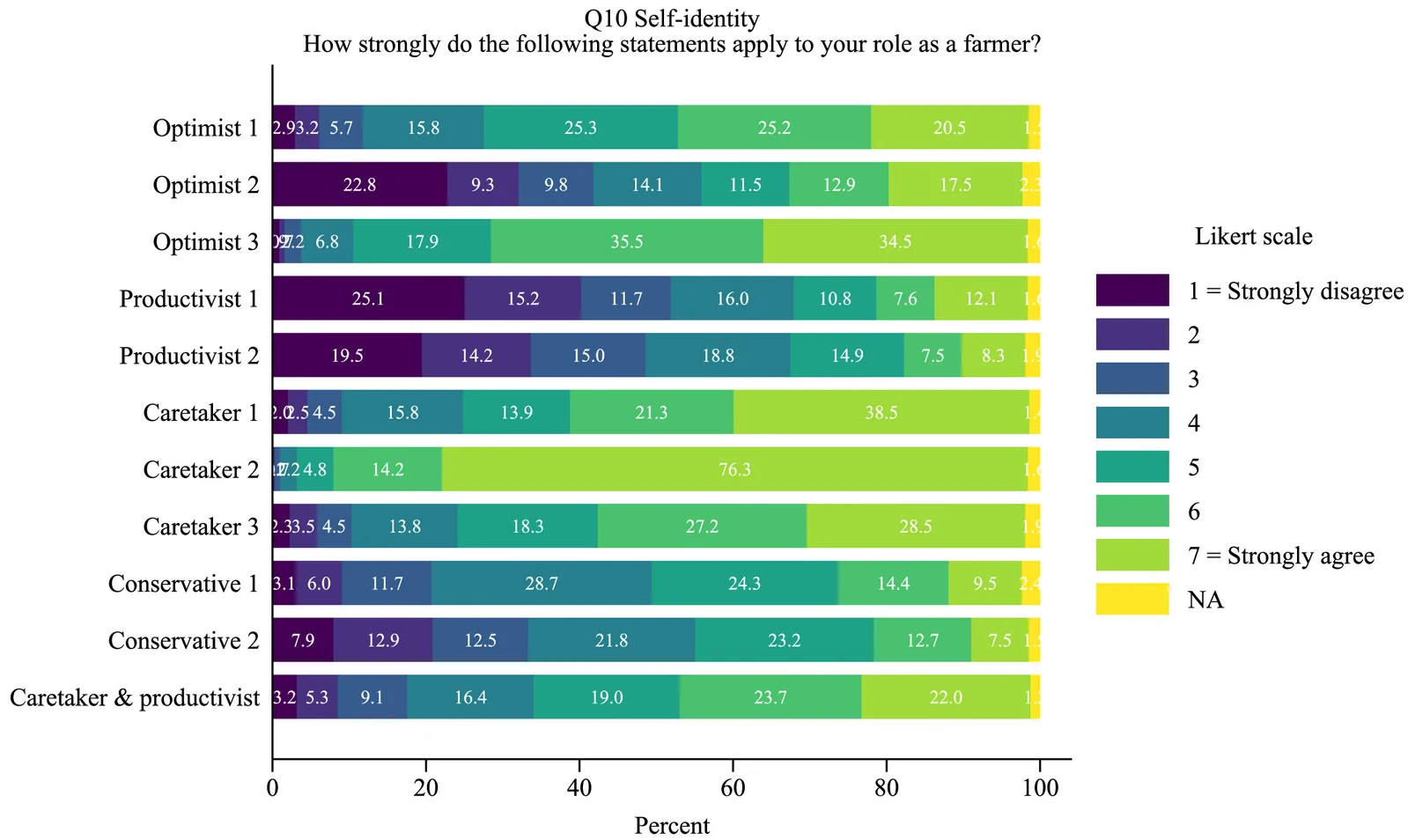
Percentage distribution of the farmers’ responses to the 11 farmer self-identity items.

### 3.4 Agricultural policy

The farmers rated the extent to which Swiss agricultural policy contributes to securing agricultural incomes (v171), ensuring national food self-sufficiency (v172), and protecting the environment and biodiversity (v173) on a 7-point Likert scale ranging from “1 = far too less” to “7 = far too much.” Fig. 6 shows the percentage distribution of the farmers’ responses to these three items, including missing values (NA). These items were developed specifically for this survey.

**Fig. 6:**
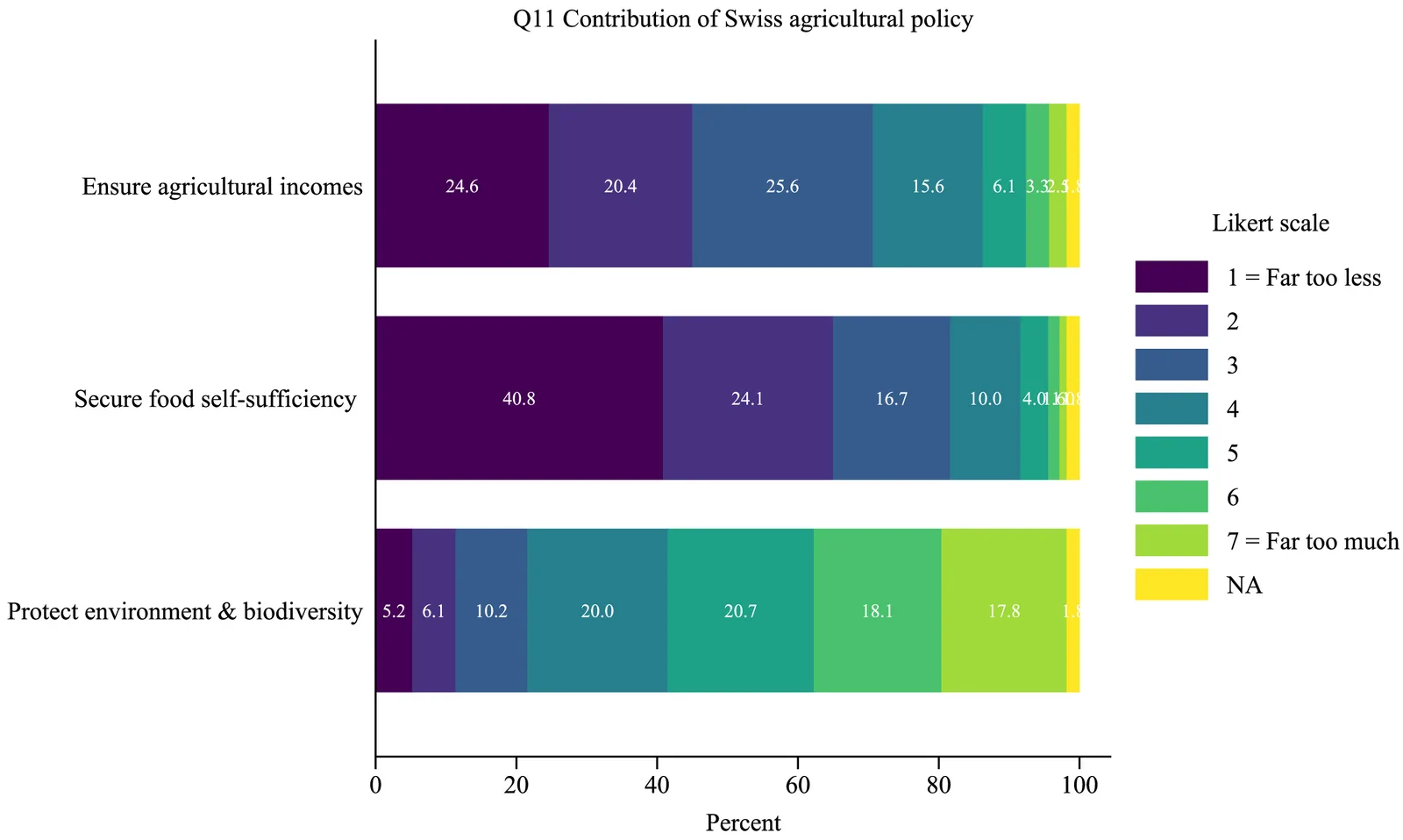
Percentage distribution of the farmers’ responses to three items reflecting the contribution of the Swiss agricultural policy.

In total, 24.6% of the farmers indicated that the Swiss agricultural policy contributes “1 = far too little” to ensuring agricultural incomes, while only 2.5% selected “7 = far too much.” Regarding food self‑sufficiency, 40.8% rated the contribution as “1 = far too little,” and only 1.0% indicated “7 = far too much.” Regarding contribution to protecting the environment and biodiversity, 5.2% of the farmers selected “1 = far too little,” whereas 17.8% selected “7 = far too much.” For all three items, missing values accounted for 1.8%.

The farmers were also asked to hypothetically allocate the federal agricultural budget according to the importance of the following seven aspects: reduce nutrient surpluses (v174), foster animal welfare (v175), secure adequate incomes (v176), reduce greenhouse gas emissions (v177), increase food production (v178), foster biodiversity (v179), reduce pesticide inputs (v180), and reduce consumer prices (v181). Fig. 7 visualizes the percentage distribution of the farmers’ responses to eight 7-point Likert scale items capturing their policy priorities, ranging from “1 = not important at all” to “7 = very important.” Missing values are indicated by NA. This set of items was adopted from El Benni et al. [8].

**Fig. 7:**
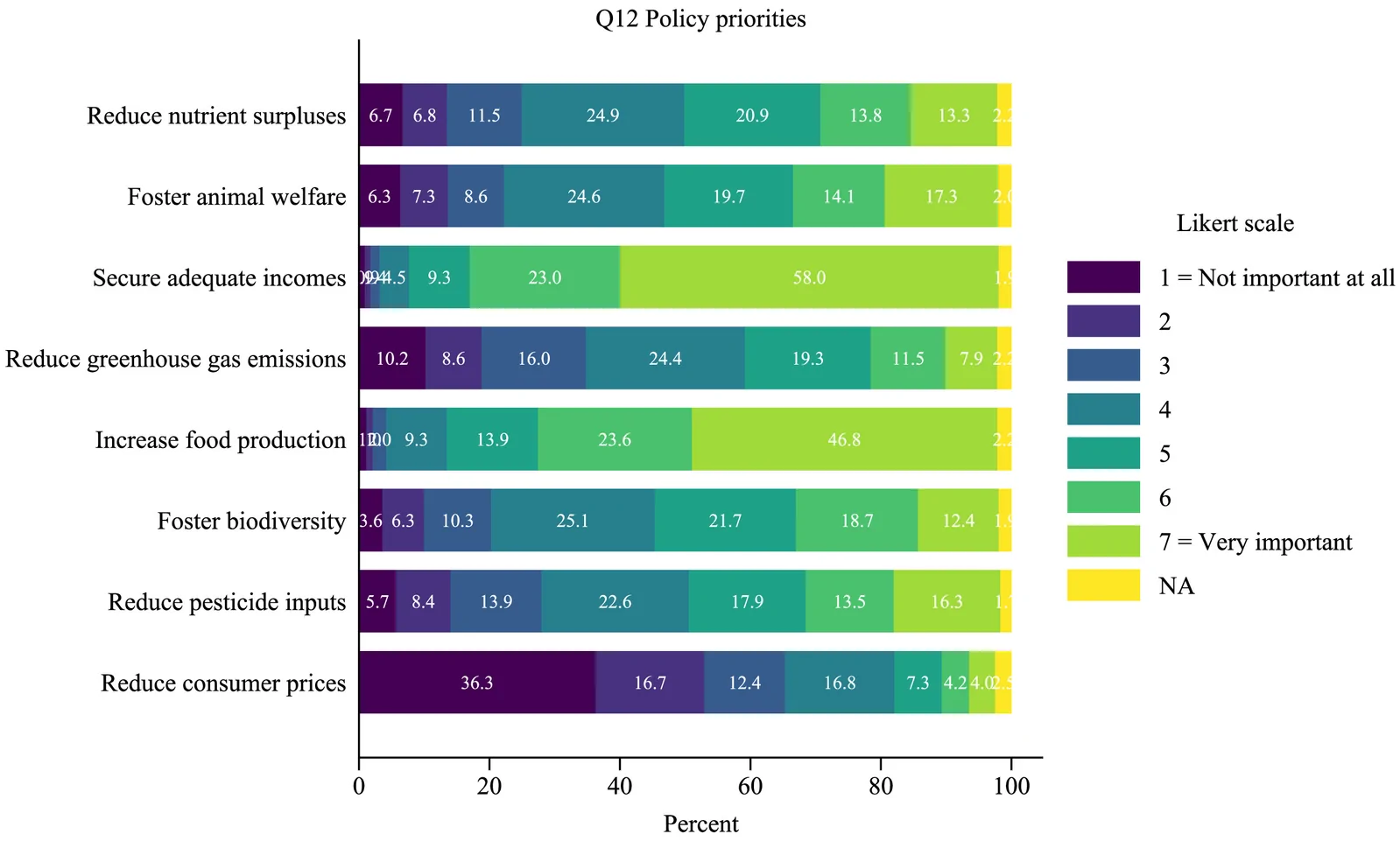
Percentage distribution of the farmers’ responses to seven policy priority items.

Regarding the objective of reducing nutrient surpluses, 6.7% of the farmers rated it as “1 = not important at all,” whereas 13.3% rated it as “7 = very important.” Fostering animal welfare was rated as “1 = not important at all” by 6.3% of the farmers, while 17.3% rated it as “7 = very important.” Only 0.9% of the farmers responded “1 = not important at all” to the item secure adequate incomes, whereas 58.0% responded “7 = very important.” Reducing greenhouse gas emissions was rated as “1 = not important at all” by 10.2% of the farmers and as “7 = very important” by 7.9%. Regarding the objective of increasing food production, 1.1% of the farmers rated it as “1 = not important at all,” whereas 46.8% rated it as “7 = very important.” Fostering biodiversity was rated as “1 = not important at all” by 3.6% of the farmers and as “7 = very important” by 12.4%. Regarding reducing pesticide inputs, 5.7% of the farmers selected “1 = not important at all,” while 16.3% selected “7 = very important.” Reducing consumer prices was rated as “1 = not important at all” by 36.3% of the farmers, whereas only 4.0% rated this objective as “7 = very important.” Missing values ranged between 1.7% and 2.5%.

Fig. 8 shows the percentage distribution of the farmers’ responses to nine 7-point Likert scale items regarding the importance of various income sources for their farms, ranging from “1 = not important at all” to “7 = very important.” Responses were provided for the following nine income sources: farm sales (v182), other on-farm business branches (v183), off-farm income (v184), off-farm income partner (v185), food security payments (v186), production system payments (v187), biodiversity payments (v188), landscape quality payments (v189), and cultural landscape payments (v190). Missing values are indicated by NA. These items were newly developed for the present survey.

**Fig. 8:**
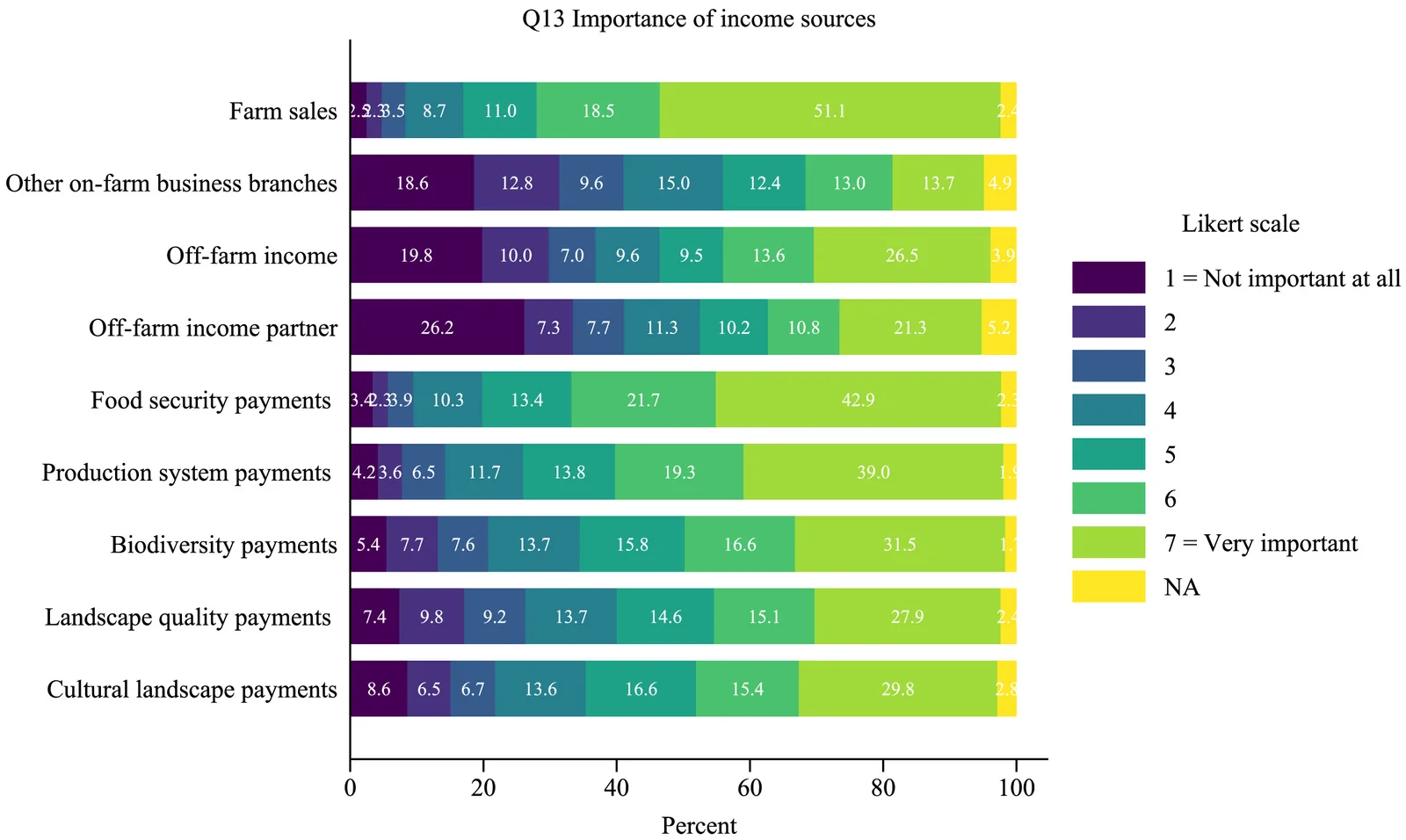
Percentage distribution of the farmers’ responses to nine 7-point Likert scale items regarding the importance of various income sources for their farms.

The importance of farm sales was rated as “1 = not important at all” by 2.5% of the farmers and as “7 = very important” by 51.1%. A share of 18.6% of the farmers indicated that other on-farm business branches were “1 = not important at all,” while 13.7% indicated “7 = very important.” Off-farm income was rated as “1 = not important at all” by 19.8% of the farmers and as “7 = very important” by 26.5%. For 26.2% of the farmers, the off-farm income of the partner was “1 = not important at all,” while 21.3% rated it as “7 = very important.” For the items capturing the importance of direct payments for farm income, the share of responses indicating “1 = not important at all” ranged between 3.4% (food security payments) and 8.6% (cultural landscape payments), whereas the share indicating “7 = very important” ranged between 27.9% (landscape quality payments) and 42.9% (food security payments).

## 4 Experimental Design, Materials, and Methods

Between June and August 2023, the survey was sent to 2000 randomly selected Swiss farmers from the German- and French-speaking regions of the country. The survey achieved a response rate of 44%, resulting in 882 observations, of which 662 were collected on paper and 220 online. Participants had the choice of entering into a prize draw for LANDI vouchers (a Swiss agricultural product retailer) to incentivize participation. Seven vouchers were given away: one worth CHF 300, one worth CHF 200, and five worth CHF 100 each. Stratified sampling was applied by agricultural zone and farm type. The farmers’ contact details were provided by the Swiss Federal Office for Agriculture, which maintains a database of approximately 48,000 farms receiving direct payments, covering over 99% of all Swiss farms. Farmers were sent a paper-and-pencil questionnaire by postal mail. The accompanying letter included an optional link for farmers who wished to complete the survey online. We sent a reminder letter by postal mail in July 2023. The questionnaire covered agricultural policy priorities, the importance of different sources of farm income, social-psychological aspects (including norms and self-efficacy), and sociodemographic characteristics. Overall, the sample closely represents the population of Swiss farms (see Fig. 9). Farms in the valley zone are slightly overrepresented, and farms in mountain zones 3 and 4 are slightly underrepresented (Fig. 9(b)).

**Fig. 9:**
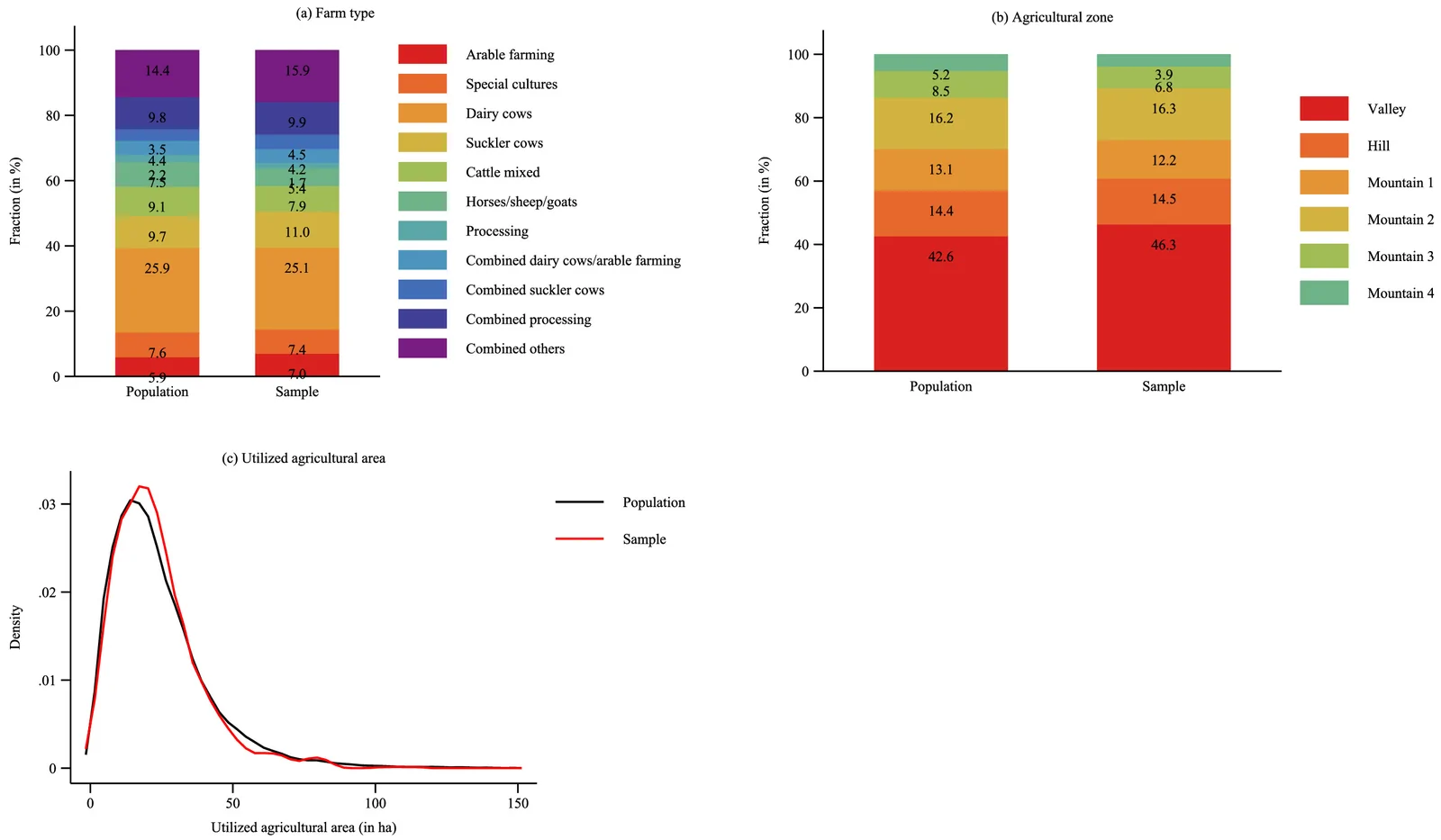
Representativeness of the sample in terms of (a) farm type, (b) agricultural zone, and (c) farm size.

## Limitations

The second part of the survey on landscape features on farms, as described in Section 3.2, could not be answered by the participants as initially intended during the survey design. The reasons for this response pattern remain unclear. One possibility is that respondents found it difficult to provide detailed assessments of landscape features based on their recollection at the time of the survey. Another is that the relatively detailed set of questions may have reduced respondents’ willingness to complete this section. These potential explanations concern the circumstances of survey completion and were not identified during pre-testing. We found no evidence of technical issues that could account for the observed pattern. As a result, data quality is limited, which is particularly reflected in a high share of missing values for variables related to aspect importance.

## Ethics Statement

The researchers adhered to all ethical considerations during the data collection process and followed institutional guidelines [9]. Participation was voluntary. The participants were assured full anonymity and data privacy in the cover letter of the survey.

## CRediT Author Statement

**Christian Ritzel:** Conceptualization, Formal analysis, Data Curation, Writing – Original Draft, Visualization, Project administration. **Antonia Kaiser:** Data Curation, Writing – Review & Editing, Project administration. **Yanbing Wang:** Data Curation, Writing – Review & Editing, Project administration. **Gabriele Mack:** Writing – Review & Editing, Supervision.

## Data Availability

ZenodoDataset on biodiversity conservation among Swiss farmers: A focus on landscape features (Original data).

## Footnotes

1 IP SUISSE represents the cooperation of Swiss farmers producing according to the guidelines of integrated agriculture [10].
